# Emerging Radionuclides in a Regulatory Framework for Medicinal Products – How Do They Fit?

**DOI:** 10.3389/fmed.2021.678452

**Published:** 2021-05-28

**Authors:** Clemens Decristoforo, Oliver Neels, Marianne Patt

**Affiliations:** ^1^Department of Nuclear Medicine, Medical University Innsbruck, Innsbruck, Austria; ^2^Department of Radiopharmaceuticals Production, Institute of Radiopharmaceutical Cancer Research, Helmholtz-Zentrum Dresden-Rossendorf, Dresden, Germany; ^3^Department for Nuclear Medicine, Radiochemistry, University of Leipzig, Leipzig, Germany

**Keywords:** radionuclides, regulatory, medicinal product, directive 2001/83, radionuclide precursor, theranostics, European Pharmacopeia

## Abstract

Recent years have seen the establishment of several radionuclides as medicinal products in particular in the setting of theranostics and PET. [^177^Lu]Lutetium Chloride or [^64^Cu]Copper Chloride have received marketing authorization as radionuclide precursor, [^68^Ga]Gallium Chloride has received regulatory approval in the form of different ^68^Ge/^68^Ga generators. This is a formal requirement by the EU directive 2001/83, even though for some of these radionuclide precursors no licensed kit is available that can be combined to obtain a final radiopharmaceuticals, as it is the case for Technetium-99m. In view of several highly promising, especially metallic radionuclides for theranostic applications in a wider sense, the strict regulatory environment poses the risk of slowing down development, in particular for radionuclide producers that want to provide innovative radionuclides for clinical research purposes, which is the basis for their further establishment. In this paper we address the regulatory framework for novel radionuclides within the EU, the current challenges in particular related to clinical translation and potential options to support translational development within Europe and worldwide.

## Introduction

Nuclear Medicine is rapidly advancing, novel targets are exploited and provide new opportunities for molecular imaging but in particular also targeted radionuclide therapy (1, 2). The driving forces clinically are advances in oncology and in this context especially Theranostics (3). The marketing authorization of the theranostic pair [^68^Ga]Ga-DOTATOC (as SomaKit TOC®) and [^177^Lu]Lu-DOTATATE (as Lutathera®) in 2017 both in Europe and the US have boosted the interest in this field also of big pharma (4), the success of PSMA inhibitors in prostate cancer has added substantially to this development (5). So far, the clinical application is dominated by the use of Gallium-68 for diagnosis using PET, to a lesser extent Indium-111 and Technetium-99m for SPECT, as well as Lutetium-177 for the therapeutic use. In Europe these radionuclides are available in pharmaceutical form as licensed products with marketing authorization, either in the form of radionuclide generators (^68^Ga and ^99m^Tc) or as radionuclide precursor formulations (^177^Lu, ^111^In, ^64^Cu, ^90^Y).

However, advances are not limited to wider use of these established radionuclides, research on theranostics also was stimulated by investigation of alternative radionuclides to improve therapeutic efficacy, to adapt the physical half-life to the target under investigation or to improve the “matched pair” concept, i.e., eliminating differences in chemistry between a diagnostic and therapeutic radionuclide (6, 7). A high interest emerged in the use of alpha emitters, to a great extent driven by the impressive results of using Actinium-225 labeled PSMA ligands, even when ^177^Lu-analogs had failed (8) with an ever increasing number of publications on radionuclide production, preclinical and clinical results (9). The development of intracellular targeted agents drives the interest in using radionuclides with a subcellular therapeutic range, in particular Auger electron emitters (10). The need for appropriate dosimetry calculation in the diagnostic application stimulated in particular the development of Positron emitters with longer half-lives such as Scandium-44 (11), this also in combination with its matched pair Scandium-47. Other matched pairs of interest are ^64^Cu/^67^Cu or the Terbium-isotopes ^149/152/155/161^Tb, even providing the possibility to combine SPECT, PET, beta and alpha therapy (12). The combination of radionuclide production with mass separation techniques may allow to obtain radionuclides in improved quality for novel applications, e.g., high specific activity ^169^Er (13). Most of these emerging radionuclides require special production techniques, such as high energies, and highly specialized infrastructure, only available in certain research institutions.

Besides such technological challenges and economic considerations, the development of these emerging radionuclides and radiopharmaceuticals thereof, in particular in the context of theranostics, is also driven by the requirement to comply with pharmaceutical regulations and guidelines, which is in particular challenging in a research setting. In this paper we address the pharmaceutical framework in relation to radionuclides and radiopharmaceutical development in Europe and finally discuss limitations and prospects related to this.

## Regulatory Framework Related to Radionuclides and Radiopharmaceuticals in Europe

In Europe medicinal products are regulated in Directive 2001/83/EC (14, 15). It defines a medicinal product as “*any substance or combination of substances presented for treating or preventing disease in human beings… and any substance or combination of substances which may be administered to human beings with a view to making a medical diagnosis*….” Radiopharmaceuticals are covered by the directive including both diagnostic and therapeutic applications, unless they are viewed as a sealed source, then the Medical Device regulation applies (16), such as in the case of SIR-Spheres (Y-90 resin microspheres, SIRTEX®). The main consequences of inclusion of radiopharmaceuticals in Directive 2001/83/EC is the requirement to marketing authorization (Article 6) and the production process according to Good Manufacturing Practices (GMP) (Article 48f), the GMP guidelines are specified in a separate Directive 2003/94/EC (17). Exemptions to the Directive exist, in particular related to pharmacy practices (18) and for investigational medicinal products (IMPs) used in clinical trials (see below). The requirements for radiopharmaceuticals within the Directive are extended toward radionuclide generators, kits and so-called radionuclide precursors. A radionuclide precursor is defined as “*Any other radionuclide produced for the radio-labeling of another substance prior to administration*.” This rather general definition includes practically all radionuclides and brings high requirements for radionuclide producers intending to supply radionuclides to a hospital or research facility for preparation of a radiopharmaceutical, which recently has been addressed in detail (19).

Besides by this central pharmaceutical directive 2001/83/EC the process of development and production of radiopharmaceuticals and radionuclides is regulated by a complementing pool of directives, regulations, guidelines and guidance documents (15). Whereas, additional directives amend the central directive with respect to specific topics, e.g., pharmacovigilance or GMP, often with no direct relation to radiopharmaceuticals or radionuclides and are released by the European Parliament and the Council, some important guidelines are coming from the European Medicines Agency (EMA). In view of radiopharmaceuticals and radionuclides they can be viewed as documents in relation to quality of the Medicinal Product or rather related to safety issues. Among other more general guidelines a central document related to quality documentation is the Guideline for Radiopharmaceuticals (EMEA/CHMP/QWP/306970/2007), defining the specific requirements for radiopharmaceuticals (also potentially including radionuclide precursors) in the application dossier for a Marketing authorization and provides some guidance to data to be included within a dossier. This can also serve as a reference e.g., in the case of a clinical trial application. A number of documents for EMA describe the format of application dossiers in relation to clinical trials or marketing authorization, as well as with specific topics e.g., requirements of validation processes. In relation to safety documentation a central document is the ICH guideline M3 (20), which defines the requirements for non-clinical safety studies that are recommended for Clinical Trial and Marketing Authorization processes. Based on discussions within the radiopharmaceutical community (21), EMA recently has released specific guidance for non-clinical requirements related to safety of radiopharmaceuticals (22) and is one of the few examples on a dedicated guidelines dealing with radiopharmaceuticals.

Apart from laws and guidelines for Medicinal products coming from the EU, the Council of Europe provides the legally binding framework of the European Pharmacopeia (see below). Also, non-binding documents from international organizations such as the Pharmaceutical Inspection Co-operation Scheme (PIC/S) or professional associations such as the European Association of Nuclear Medicine (EANM) complement with legally non-binding, but more specific documents the pharmaceutical regulations of radiopharmaceuticals and radionuclides in Europe, examples thereof are found in the following chapters and an overview of legally binding and guidance documents is given in Table 1.

**Table 1:** Overview of legally binding and guidance documents for radiopharmaceuticals.

	**Origin**	**Aim/Content**	**Legal status**
Directive 2001/83/EC	EC	Community Code directive, establishment of the basic principles for manufacture and marketing of medicinal products in the EU	Legally binding after transfer into national law
Directive 2001/20/EC	EC	Harmonization of the requirements for the conduct of Clinical Trials in the EU, introduction of EudraCT database, adoption of GCP rules	Legally binding after transfer into national law
Directive 2003/94/EC	EC	Obligation of manufacturers to comply with the principles of GMP for production of medicinal products	Legally binding after transfer into national law
Guideline for Radiopharmaceuticals (EMEA/CHMP/QWP/306970/2007)	EMA	Requirements for radiopharmaceutical for obtaining marketing authorization for the European single market	Guidance document by European authority, not legally binding
Guideline on the non-clinical requirements for radiopharmaceuticals (EMA/CHMP/SWP/686140/2018)	EMA	Requirements for preclinical safety testing of radiopharmaceuticals	Guidance document by European authority, not legally binding
Regulation EU 536/2014	EC	Improvement of administrative requirements for the conduct of clinical trials in the EU	Immediately binding for EU member states
Guide to Good Manufacturing Practices of preparation of medicinal products in healthcare establishments	PIC/S	Description of the requirements for in house production of medicinal products in hospitals and other healthcare establishments	Guidance document by international pharmaceutical association, not legally binding
ICH guideline M3 (R2)	ICH	Requirements for non-clinical safety studies for the conduct of human clinical trials and marketing authorization for pharmaceuticals	Guidance document by European and non-European authorities, not legally binding
Monographs of the European Pharmacopeia	EDQM, Council of Europe	Monographs for medicinal products, reagents and starting materials	Legally binding
General text 5.19 on Extemporaneous preparation of radiopharmaceuticals of the European Pharmacopeia	EDQM, Council of Europe	Description of the requirements for extemporaneous, non-industrial preparation of radiopharmaceuticals	Guidance document by European institution, not legally binding
Guideline on current good radiopharmacy practice (cGRPP) for the small-scale preparation of radiopharmaceuticals	EANM	Description of the requirement for small-scale, non-industrial preparation of radiopharmaceuticals	Guidance document by professional society, not legally binding
EANM guideline for the preparation of an Investigational Medicinal Product Dossier (IMPD)	EANM	Structure and content of an IMPD for radiopharmaceuticals	Guidance document by professional society, not legally binding

*EC, European Commission; EMA, European Medicines Agency; EDQM, European directorate for quality of medicines; ICH, International Council for Harmonization of Technical Requirements for Pharmaceuticals for Human Use; GCP, Good clinical practice; EANM, European Association of Nuclear Medicine*.

## Radionuclides and GMP

Medicinal products must be prepared according to GMP, the GMP guidelines of the EU have legal status as stated both for medicinal products in directive 2001/83 (23) and IMPs in the clinical trials directive 2001/20/EC (24). Specific reference to radiopharmaceuticals is given in Annex 3 on “Manufacture of Radiopharmaceuticals,” however, with little specific guidance to radionuclide production or quality control. The term “radioactive precursor” is used and it remains inconclusive whether this term covers radionuclides in general or only “ready for use” radionuclide precursors. A clear statement is included that for cyclotron or reactor production GMP is not applicable, whereas processing, purification and formulation are to be taken into account. This perspective for manufacturing may, however, not always meet specific requirements especially for small scale preparation settings (18). For this dedicated practice guidance for a suitable quality framework has been released. PIC/S has published a dedicated Guidance for Good practices in Healthcare establishment (25) with a separate Annex to radiopharmaceuticals, referring to radionuclides also produced on site via cyclotron, interestingly also mentioning the use of radionuclides being supplied as radiochemicals for preparation of radiopharmaceuticals. Another guidance for an appropriate quality framework was released by EANM (26), however without specific additional relations to radionuclides in particular. Recently, a Chapter on “Extemporaneous preparation of radiopharmaceuticals” (27) has been published within the European Pharmacopeia (see below). Even though chapters are not legally binding, it provides guidance on the quality framework of small-scale radiopharmaceutical preparations and contains a dedicated sub-chapter on production of radionuclides and e.g., how to ensure the quality of a target material. Overall, the guidance on how to ensure a suitable quality framework in the context of production and quality control of novel radionuclides in particular is rather scarce.

## Radionuclides and Clinical Trials

To date most novel theranostic radionuclides have not been applied, yet, within prospective controlled Clinical Trials. Clinical Trials are strictly regulated within the EU, but also internationally (28). Within the EU currently the Clinical Trials Directive 2001/20/EC is in force, that should be replaced by the new Regulation 536/2014 (29). This new regulation provides an exception from GMP compliance for diagnostic radiopharmaceuticals, which may have an impact also on the use of novel radionuclides, albeit, not in the therapeutic setting (30). To initiate a clinical trial with novel radionuclides requires generation of data both on the quality of the radionuclide as well as on its safety, to address the requirements for the submission dossier of the IMP, i.e., the radiopharmaceutical under investigation. This investigational Medicinal Product Dossier (IMPD) describes on the one hand the chemical and pharmaceutical quality documentation. For this part detailed guidance is given and also specifically been provided for radiopharmaceuticals (31). For the radionuclide the production route, decay characteristics and quality aspects have to be described. On the other hand, the IMPD needs to contain data on the safety, including pharmacology and toxicology as well as efficacy, if available. Regarding radionuclides, pharmacology, of course is dependent on the radiopharmaceutical itself. Related to toxicity, a recent draft guideline describes specific requirements for the so called non-clinical safety of radiopharmaceuticals (22), stating the requirements for the non-radioactive part of a radiopharmaceuticals. The challenges in fulfilling toxicity requirements for radiopharmaceuticals have recently been summarized (21). It clearly excludes definitions related to radiation related toxicity, which is covered by respective radiation protection guidelines. However, a main part of the IMPD addresses the risk benefit analysis of a new radiopharmaceutical and with it a novel radionuclide that is applied. The risk analysis, of course, has to take into account the potential radiation damage induced by application of the radiopharmaceutical, which has to be derived from dosimetry studies. Therefore, dosimetry data need to be included in an IMPD. Detailed discussion on this topic is out of scope of this paper and requires separate in-depth discussion (32). Overall, the submission of a dossier for a clinical trial involving a novel radionuclide requires extensive data compilation, whereby only limited guidance is available from the regulatory side.

## Radionuclides and the European Pharmacopeia

The European Pharmacopeia [Pharm Eur, most recent 10th edition (33)] releases monographs and chapters related to the quality of medicines. It is in the responsibility of the European Directorate for quality of Medicines (EDQM), which is a body of the Council of Europe. Within the “Convention on the Elaboration of a European Pharmacopeia” member states agree to implement Pharm Eur in their national drug regulation, thereby monographs become legally binding documents. The convention covers a wider range of countries, not only the European Union.

Pharm Eur has a number of monographs specifically dealing with radiopharmaceuticals. In contrast to chemical precursors, where a dedicated monograph exists, there is no monograph dedicated to radionuclides or radionuclide precursors. However, the general monograph “Radiopharmaceutical preparations” (monograph 0125) specifically also includes radionuclide precursors and provides general definitions and tests including e.g., radionuclidic and radiochemical purity testing as well as specific provisions for sterility and bacterial endotoxin testing. A table of “Physical Characteristics of Radionuclides mentioned in the European Pharmacopeia” complements this monograph, however, does not include emerging novel radionuclides, such as Copper, Scandium or Terbium isotopes. A dedicated chapter on “Detection and Measurement of Radioactivity” provides the pharmaceutical view on specific radioanalytical methods for radiopharmaceuticals and radionuclides.

A number of specific radionuclide precursor monographs (containing “for radiolabelling” in the title) are available for established radionuclides such as Fluorine-18, Iodine-123 and −131, or Indium-111. More recently Lutetium (^177^Lu) solution for radiolabelling (monograph 2798) and Yttrium (^90^Y) chloride solution for radiolabelling (monograph 2803) as therapeutic radionuclides were added. For Gallium-68 even 2 monographs are available Gallium (^68^Ga) chloride solution for radiolabelling (monograph 2464) and Gallium (^68^Ga) chloride (accelerator-produced) solution for radiolabelling (monograph 3109). These monographs describe the quality requirements for these radionuclides. The challenges in defining these are manifold. First, the quality of the radionuclide is also dependent on the production route and especially radionuclidic impurities or impurity levels can be completely different. This is, e.g., reflected in the two aforementioned Gallium-68 monographs. Whereas, the “traditional” route of generator production requires definition of a limit for Germanium-68, in the case of accelerator-produced pathway radionuclidic impurities are mainly Gallium-66 and−67. Second, the quality is also dependent on the actual radiopharmaceutical being prepared from a certain radionuclide. E.g., the administered activity may vary considerably dependent on whether a long circulating antibody is labeled or a small, rapidly excreted peptide is applied, even in several administrations. The radionuclidic impurity, most likely, will biologically behave in the same manner. Therefore, a certain limit calculated for a certain application may not necessarily be valid for another and has to be viewed on a case-by-case basis. Similar considerations apply e.g., to the chemical purity of a radionuclide. If the molar activity of a certain radiopharmaceutical to be labeled with a radiometal in question has to be high, impurity levels for interfering metals should be low. This also depends on the chemistry, or, more precisely, the chelator used for attachment of a radiometal. A chelator, which is highly specific for a certain radiometal, may allow for higher impurity levels then a more general chelator such as DOTA. Other quality requirements can also not be generalized. E.g., testing for sterility of a radionuclide may not be required if the production process ensures suitable removal of microbiological contamination, the same is true for endotoxins. However, the existing monographs already provide a good basis of understanding on how to define quality requirements also for novel radionuclides intended for radiopharmaceutical preparations. A good overview on this topic can also be found in a recent publication on Radiopharmaceutical Precursors for Theranostics (34).

## Outlook

Introducing novel radionuclides into clinical practice is a challenging process from a pharmaceutical regulatory perspective. Good practices should be followed in this process including GMP, GDP, GLP and GCP (35). The EU has set a number of initiatives to ensure the development of novel radionuclides including the SAMIRA study (36) or by support of infrastructure projects such as CERN-MEDICIS (37). Another initiative aimed at identification of methods and technologies as well as supply of medical radioisotopes in use and expected to be in use by 2030 in support of Europe's beating cancer plan (38). However, efforts to provide support in relation to the pharmaceutical regulatory framework are scarce. The translation of novel radionuclides and with them respective novel radiopharmaceuticals especially for theranostics has to be performed within the constraints of this framework, which remains one of the major challenges. Initiatives to provide guidance for radiopharmaceuticals have been made by professional organizations such as the EANM with e.g., dedicated guidelines for good practices in the small scale preparation of radiopharmaceuticals (39). A recently granted EU project (PRISMAP) brings together many non-profit centers with the infrastructure and know-how in the production of novel radionuclides including high energy accelerators, reactors and mass separation facilities with the aim to provide a stable basis for future supply of innovative radionuclides. This project also includes the aim to provide standards for the clinical translation of such radionuclides also to comply with pharmaceutical regulations. This support by the European Union both for the infrastructures of radionuclide production sites and their collaboration, should, however, be accompanied by a suitable regulatory framework, that supports innovation without impairing the safety within medical applications. In this context and considering the great potential of theranostics, which depends on the application of novel radionuclides, requires adaptation of legislation and supporting guidelines to the current state of the art, e.g., including appropriate legal definition of radionuclides used for medicinal products (19). An intensive discussion and close collaboration between radionuclide producers, researchers developing novel radiopharmaceuticals, professionals in Nuclear Medicine departments and regulators from European and national pharmaceutical authorities is required to ensure not only the supply of novel radionuclides but also their clinical applicability.

## Author Contributions

CD, MP, and ON have written the draft manuscript. MP and ON provided tables and CD did final editing of the manuscript. All authors contributed to the article and approved the submitted version.

## Conflict of Interest

The authors declare that the research was conducted in the absence of any commercial or financial relationships that could be construed as a potential conflict of interest.
